# Maternal and Perinatal outcome after Ramadan Fasting

**DOI:** 10.12669/pjms.36.5.2612

**Published:** 2020

**Authors:** Rashida Parveen, Mehnaz Khakwani, Munazza Latif, Ayesha Uzaima Tareen

**Affiliations:** 1 Rashida Parveen, Department of Obstetrics and Gyne, Unit-II, Nishtar Medical University & Hospital, Multan, Pakistan; 2 Mehnaz Khakwani, Department of Obstetrics and Gyne, Unit-II, Nishtar Medical University & Hospital, Multan, Pakistan; 3 Munazza Latif, Department of Obstetrics and Gyne, Unit-II, Nishtar Medical University & Hospital, Multan, Pakistan; 4 Ayesha Uzaima Tareen, Department of Obstetrics and Gyne, Unit-II, Nishtar Medical University & Hospital, Multan, Pakistan

**Keywords:** Fasting, Maternal, Perinatal, Outcome

## Abstract

**Objective:**

To determine maternal and perinatal outcome after Ramadan fasting during pregnancy

**Methods:**

This cross sectional study was conducted at The Department of Obstetrics and Gynaecology, Nishtar Hospital, Multan from May to October 2019. A total of 226 women attending labour room, aged 18–35 years, having gestational amenorrhoea 15 – 40 weeks of gestation were included. Women who fasted for more than 15 days were compared with those who did not fast or fasted for less than 15 days in the month of Ramadan. Demographical profile along with maternal and perinatal outcomes were compared between the study groups considering p value less than 0.05 as significant.

**Results:**

Out of 226 women, 58 (25.7%) fulfilled the criteria to be included in the fasting group while remaining 168 (74.3%) were slotted in the non-fasting group. There was no difference (p value > 0.05) in between the both group with respect to demographical characteristics except significantly less women were employed in the fasting group (p value=0.0246). No statistical difference was found in terms of maternal or perinatal outcomes between both the study groups.

**Conclusion:**

Fasting women were not found to have poor maternal and fetal outcomes when compared to non-fasting women.

## INTRODUCTION

Muslims fast during the month of Ramadan and fasting is compulsory in their religion.^1^ They fast from sunrise to sunset during the month of Ramadan which is the 9^th^ lunar month, so the duration of fast can vary from 13 to 18 hours per day. During fast, they avoid drinking and eating anything.^2^ Although fasting is difficult during pregnancy but many pregnant Muslim women choose to fast during the month of Ramadan.^3^

Maternal malnutrition and fasting can affect maternal metabolism.^4^ If a woman fasts for 13 hours or more, corticotrophin releasing hormone is increased in maternal serum as compared with those women who fast for less than 13 hours. Corticotropin releasing hormone is a stress hormone and associated with preterm delivery and fetal growth restriction.^5^ Due to fasting blood sugar levels can decline leading to changes in lipid metabolism and subsequent increasing levels of blood ketones.^5^ This can result in decreased blood pH or Ketoacidosis.^6^ In all the studies about Ramadan fasting during pregnancy, the results are contradictory but most of them have concluded that Ramadan fasting does not have significant effect on Perinatal outcomes.^7^

Many Muslim women seek advice from health practitioners regarding the safety of Ramadan fasting in pregnancy.^8^ However the current information available lacks clear guidelines and we do not have such studies in our region. Here in Multan, the climate in month of Ramadan is hot and duration of fast is long.^9^ In a study, 38.7% of women completed fasting for the entire Ramadan period and fasting during the second trimester of pregnancy with decrease in the risk of gestational diabetes.^10^ By keeping in mind the controversies, the aim of this study was to determine maternal and perinatal outcome after Ramadan fasting during pregnancy so that we can guide our pregnant women about Ramadan fasting.

## METHODS

This cross sectional study was conducted at The Labour Room of Department of Obstetrics and Gynaecology, Nishtar Hospital, Multan from May to October 2019. Approval from Institutional Ethical Committee was taken (Ref. No. 4000, dated 19-02-2020).

Using WHO calculator, sample size was 226. The calculations of sample size were based on the given proportion of pregnancy outcomes, 38.7% women completed fasting for the entire month of Ramadan.^10^ At 95% confidence interval and 90% power of the test. Total 226 cases would be required to conduct the study. Using non probability consecutive sampling, a total of 226 women, aged 18-35 years, having gestational amenorrhoea 15 – 40 weeks of gestation were included. Informed consent was taken from all the study participants. Women with pre-existing diabetes, chronic hypertension, endocrine disorders, gastrointestinal disorders, malabsorption syndromes, history of renal disease, gall stones or those unsure of last menstrual period were not enrolled. Those women who lost follow up were also excluded.

The period of Ramadan was from 5^th^ May to 4^th^ June in 2019 and fasting times on an average up to 15 hours per day. Initially asked question from all women was that did they fast in the course of their current pregnancy or not. Then women in the fasting were asked for the number of days they had fasted, which was categorized more than 15 days or less than 15 days. Women who fasted for more than 15 days were compared with those who did not fast or fasted for less than 15 days in the month of Ramadan. All the study information was recorded on a special proforma containing maternal characteristics like mother’s age, BMI, gestational amenorrhea, gravid and parity status, mother’s literacy, employment status, family income along with maternal outcomes like mode of delivery, gestational diabetes, pre-eclampsia, preterm labour and perinatal outcomes like birth weight, height of baby, head circumference, mid arm circumference and weight of placenta. Hemoglobin level and random blood sugar were also measured and noted.

The data was entered and analyzed in Statistical Package for Social Sciences (SPSS), version 22. Mean ± Standard deviation were obtained for continuous variables like mother’s age (years), fetal birth weight (kg), fetal height (cm) and fetal head circumference (cm). Similarly, frequencies and percentages were obtained for categorical variables like mode of delivery, gestational diabetes, hypertension and preterm labour. Frequencies and percentages were calculated and chi-square test was performed to assess the relationship of qualitative variables while independent sample t-test was applied to quantitative variables to compare both study groups. P value less than 0.05 was considered as significant.

## RESULTS

As per inclusion and exclusion criteria, out of 226 women, 58 (25.7%) fulfilled the criteria to be included in the fasting group while remaining 168 (74.3%) were slotted in the non-fasting group. Table-I shows the characteristics of women in the both groups.

**Table-I:** Characteristics of Women in between Study Groups (n=226).

Characteristics		Group-A (n=58)	Group-B (n=168)	P-value
Mother’s Age (Mean±SD)		28.38±4.14	28.11±3.94	0.6574
Mother’s BMI (Mean±SD)		25.71±3.75	25.89±3.96	0.7626
Trimester	Second	16 (27.6%)	38 (22.6%)	0.4444
	Third	42 (72.4%)	130 (77.4%)	
Gravida Status	1-2	45 (77.6%)	128 (76.2%)	0.8287
	>2	13 (22.4%)	40 (23.8%)	
Parity Status	Nulliparous	6 (10.3%)	24 (14.3%)	0.4457
	Multiparous	52 (89.7%)	144 (85.7%)	
Mother’s Literacy	Illiterate	22 (37.9%)	35 (20.8%)	0.0694
	Primary	13 (22.4%)	54 (32.1%)	
	Secondary	12 (20.7%)	37 (22.0%)	
	Matric or Above	11 (19.0%)	42 (25.0%)	
Employed Women	Yes	8 (13.8%)	48 (28.6%)	0.0246
	No	50 (86.2%)	120 (71.4%)	
Family Income	Low	29 (50.0%)	97 (57.7%)	0.5860
	Medium	20 (34.5%)	50 (29.8%)	
	High	9 (15.6%)	21 (12.5%)	
Hemoglobin, g/dl (mean±SD)		9.52±0.71	9.72±0.88	0.1194
Random Blood Sugar, mg/dl, (mean±SD)		112.82±9.41	115.92±12.28	0.0811

There was no difference (p value > 0.05) in between the both group with respect to Mother’s age, BMI, trimester, gravid and parity status, mother’s literacy and family income whereas more women, 48 (28.6%) in the non-fasting group were employed in comparison to 8 (13.8%) in the fasting group (p value=0.0246). No statistical difference was seen among gender of the newborns between the both groups.

Table-II shows that when maternal outcomes like mode of delivery, gestational diabetes, pre-eclampsia and preterm labour were compared between fasting and non-fasting groups, no significant difference was found among the groups (p value>0.05).

**Table-II:** Maternal outcome in between Study Groups (n=226).

Maternal Outcome		Group-A (n=58)	Group-B (n=168)	P-value
Mode of Delivery	Vaginal	27 (46.6%)	97 (57.7%)	0.1399
	Cesarean	31 (53.4%)	71 (42.3%)	
Gestational Diabetes	Yes	3 (5.2%)	14 (8.3%)	0.4313
	No	55 (94.8%)	154 (91.7%)	
Pre-Eclampsia	Yes	7 (12.1%)	19 (11.3%)	0.8758
	No	51 (87.9%)	149 (88.7%)	
Preterm Labour	Yes	4 (6.9%)	9 (5.4%)	0.6642
	No	54 (93.1%)	159 (94.6%)	

Perinatal outcome in between both the study group is highlighted in Table-III. It was found that there was no significant difference in terms of perinatal outcomes like birth weight, height of baby, head circumference, mid arm circumference or weight of the placenta in between both the study groups (p value>0.05).

**Table-III:** Perinatal outcome in between Study Groups (n=226).

Perinatal Outcome	Group-A (n=58)	Group-B (n=168)	P-value
Birth Weight in Kg (Mean±SD)	2.74±0.51	2.79±0.59	0.5657
Height of Baby in cm (Mean±SD)	45.24±2.98	45.74±2.66	0.2329
Head Circumference in cm (Mean±SD)	33.68±1.48	33.63±1.38	0.8156
Mid Arm Circumference in cm (Mean±SD)	10.42±0.96	10.36±0.82	0.6465
Weight of Placenta in g (Mean±SD)	538.45±86.52	546.25±88.49	0.5611

## DISCUSSION

During Ramadan, a type of intermittent fasting is observed while quality as well as quantity of the food eaten is amended. It is important to describe that Muslim women are exempted from fasting as per the religion but data from around the world suggests that round 90% of the women fast for at least some period in the holy month of Ramadan to have that religious experience with them.^11^-^13^ Globally, an estimated 230 million Muslim adult women are fasting for at least some period in the month of Ramadan. This study was done to whether maternal or perinatal outcomes are affecting by fasting.^14^

In the present study, mean age of the women in fasting group was 28.38±4.14 years in comparison to 28.11±3.94 years in non-fasting group. These findings were pretty similar to what was found in another local study where mean age of the fasting and non-fasting group were 27.16±4.27 and 27.36±4.92 years respectively.^15^ No difference in demographical characteristics of both study groups observed noted in the present study except significantly less women in the fasting group were employed. Our findings were consistent with those of Safari K et al from Iraq^10^ where the researchers noted only 11.0% of the women to be working women in the fasting group in comparison to 20.8% in the non-fasting group and the difference was found statistically significant. This could be self-explanatory as most of the working women are exposed to excessive temperature and working environment which could be the reason that they are opting not to fast significantly more often than those who are not employed and spending most of their time at homes.

In the present study, we could not record any significant difference in terms of maternal or perinatal outcomes in between the both study group. A study from Iran by Ziaee V et al.^16^ had noted similar findings between fasting and non-fasting 189 women where no difference in maternal as well as perinatal outcomes were recorded. They did not note any inappropriate effect in terms of intrauterine growth as well as birth time indices which is quite similar to what was noted in the present study. Moradi M from Iran^17^ comparing fetal growth indices between fasting and non-fasting groups noted no significant difference in terms of fetal growth indices, amniotic fluid index or uterine and umblical indices. These findings are again emphasizing that fasting is not altering maternal or fetal outcomes. Being a neighboring country, Iran shares similar environmental and seasonal factors to Pakistan so our findings stand well correlated with those of previous researchers.

In the present study, 6.9% of the women in fasting group had preterm labour in comparison to 5.45% in the non-fasting group. Pre-term delivery or low birth weight can be a result of poor maternal nutritional status and hypothetically, Ramadan could contribute to these observations but in the present study we did not find evidence of increased preterm births or poor perinatal outcomes. A prospective study comprising of 400 women found that Ramadan fasting did not alter the birth weight of newborn.^18^ A study from Turkey^19^ comparing fasting and non-fasting women found no worse fetal outcomes between the both groups. Very similar prevalence of low birth weight babies (2.5% in each groups) were found. Similar observations are reported by other researchers as well.^15^ It is advisable that pregnant ladies who wish to fast should consult a healthcare professional before the start of Ramadan. They need to be advised to have sufficient caloric and fluid intake before and after the fasting period.^20^

Multan is considered to be one of the hottest cities of Pakistan and this study is thought to provide our perspective about the maternal and perinatal outcomes among women fasting. Similar characteristics of women among fasting and non-fasting group adds to the strength of this research.

### Limitations of this study

BMI at study entry and weight gain during fasting period, in relation to various BMI groups could not be calculated as we did not enroll all women before the month of fasting. Correlation of BMI and different gestational age groups could not be analyzed as not all the women were enrolled solely during the month of Ramadan as this was a six months duration study. Further studies are required to see the effect of various maternal and dietary factors during and after Ramadan fasting on the maternal and perinatal outcomes. Another limitation is that as information was gathered at the time of delivery, there could have been limitations to recall. We also did not compare studied outcomes in between different trimesters.

## CONCLUSION

Fasting women were not found to have poor maternal and fetal outcomes when compared to non-fasting women. More studies having different sets of populations and randomized design will further help in determining the maternal and fetal outcomes related to fasting women.

### Authors’ Contribution

**RP:** Conceived, Data Collection, Drafting, Responsible for Data integrity and authenticity.

**MK:** Supervision, Proof Reading.

**ML:** Literature Review, Discussion.

**AUT:** Data Collection, Data Analysis.
